# A Histologic, Histomorphometric, and Immunohistochemical Evaluation of Anorganic Bovine Bone and Injectable Biphasic Calcium Phosphate in Humans: A Randomized Clinical Trial

**DOI:** 10.3390/ijms24065539

**Published:** 2023-03-14

**Authors:** Matej Tomas, Matej Karl, Marija Čandrlić, Marko Matijević, Martina Juzbašić, Olga Cvijanović Peloza, Ana Terezija Jerbić Radetić, Davor Kuiš, Bruno Vidaković, Zrinka Ivanišević, Željka Perić Kačarević

**Affiliations:** 1 Department of Dental Medicine, Faculty of Dental Medicine and Health Osijek, J. J. Strossmayer University of Osijek, 31 000 Osijek, Croatia; 2 Interdisciplinary University Study of Molecular Biosciences, J. J. Strossmayer University of Osijek, 31 000 Osijek, Croatia; 3 Faculty of Medicine Osijek, J. J. Strossmayer University of Osijek, 31 000 Osijek, Croatia; 4 Community Healthcare Center of Osijek-Baranja County, 31 000 Osijek, Croatia; 5 Department of Anatomy, Faculty of Medicine, University of Rijeka, 51 000 Rijeka, Croatia; 6 Department of Periodontology, Faculty of Dental Medicine Rijeka, Univeristy of Rijeka, 51 000 Rijeka, Croatia; 7 Clinical Hospital Center Rijeka, 51 000 Rijeka, Croatia; 8 Department of Anatomy, Histology, Embriology, Pathology Anatomy and Pathology Histology, Faculty of Dental Medicine and Health Osijek, J. J. Strossmayer University of Osijek, 31 000 Osijek, Croatia

**Keywords:** alveolar bone regeneration, anorganic bovine bone, biphasic calcium phosphate, histology, immunohistochemistry

## Abstract

Following trauma, chronic periapical process, or tooth extraction, a large loss of bone volume is noticed during the healing process. To facilitate the placement of dental implants, various surgical procedures are used for an optimal alveolar ridge profile, while maintaining adequate bone dimensions. The main aim of this study was to determine the healing ability (histologically and immunohistologically) of alveolar bone defects during augmentation with two different biomaterials: injectable biphasic calcium phosphate (BCP) and anorganic bovine bone (ABB). Thirty-eight subjects were randomly divided into two groups. The first group received the tested bone substitute biomaterial (BSB), i.e., BCP (maxresorb inject^®^), and the second group received an alternative to the gold standard, i.e., ABB (Bio-Oss^®^). The histopathological, histomorphometric, and immunohistochemical analyses gave comparable results for these bone substitute materials in terms of newly formed bone: (BCP: 39.91 ± 8.49%, ABB: 41.73 ± 13.99%), residual biomaterial (BCP: 28.61 ± 11.38%, ABB: 31.72 ± 15.52%), and soft tissue (BCP: 31.49 ± 11.09%, ABB: 26.54 ± 7.25%), with no significant difference found between the groups (*p* < 0.05, *t*-test), proving that BCP is equally suitable and successful for alveolar bone regeneration.

## 1. Introduction

Following trauma, chronic periapical process, or tooth extraction, a large loss of bone volume is noticed during the healing process [1,2]. Two-thirds of bone loss occurs on the vestibular side, and most alveolar changes in the extraction socket occur in the first year after tooth extraction [3,4]. In the first six months after tooth extraction, horizontal bone loss of 29–63% and vertical bone loss of 11–22% have been reported in previous human studies [5,6,7]. However, the ultimate goal of implant therapy is to restore missing teeth by positioning the implant in an anatomically correct, aesthetically pleasing, and functional location [8]. To facilitate the placement of dental implants, various surgical procedures are used for an optimal alveolar ridge profile, while maintaining adequate bone dimensions [9,10]. Alveolar ridge preservation is described as “any method before or after tooth extraction that aims to limit ridge resorption and promote bone growth within the alveolus“; as such, it has attracted the interest of many researchers. Alveolar bone regeneration provides many different biomaterials (BSB), such as autogenous bone from oral and extra oral sites, allografts, xenografts, and synthetic biomaterials [11,12,13]. The main function of the BSB is to provide mechanical support and stimulate bone regeneration, with the ultimate goal of creating new bone [14]. In addition, BSB must reduce the risk of biological side effects [15]. Furthermore, BSB must prevent the breakdown of the structural scaffold for bone formation and preferably be replaced by newly formed bone through bone resorption and remodeling by osteoclasts [16,17,18,19]. According to studies, all current BSBs reach only the osteoconductivity requirement and instead serve as a structural scaffold for regenerative processes [20,21,22]. None of the products available today have all the characteristics that make them ideal for BSBs, such as nontoxicity, ease of handling, low immunogenicity, affordability, ability to induce blood vessel growth, biocompatibility, and osteoinductive and osteoconductive properties [14,23]. Because it remains the only marrow for healing that possesses all four of the critical biological characteristics of bone, autogenous bone is considered the gold standard in the therapeutic use of bone augmentation [24,25]. However, autografts also have a number of disadvantages. During augmentation and remodeling, autogenous bone tends to lose up to 60% of its volume [26]. Due to additional disadvantages such as secondary surgical site, limited availability, bleeding risk, edema, postoperative pain, and increased surgical costs, BSBs have been developed as an alternative [27,28,29,30,31,32,33,34]. An allogeneic bone graft is derived from an individual belonging to the same species but with a different genotype. The avoidance of a secondary surgical site and a shortened procedure time are the advantages of such BSB. Several risks associated with such BSB can be mitigated by tissue processing, such as sterilization, ultrasonic cleaning, gamma irradiation, and demineralization [35]. In addition, increasing regulatory restrictions on the use of allografts in Europe have led to the production of new materials of other origins, such as animal or synthetic [36,37]. 

Xenografts are used as an alternative to the gold standard. Most scientific research mentions deproteinized bovine bone, porcine bone, and more recently, horse bone [38]. Deproteinized bovine bone is the most typical source of xenografts in dentistry. To produce a porous hydroxyapatite (HA) material containing only the anorganic components of bovine bone, the bone is either thermally deproteinized and/or chemically processed e.g., NaOH. The resulting porous structure can provide strong mechanical support and promote healing. The porous structure also has a large surface area and promotes angiogenesis, i.e., the formation of new blood vessels. Several studies have shown that HA is fully integrated into regenerated bone. Studies have shown that the risk of disease transmission is minimal, despite the suspected possibility of organic residues in bovine bone substitutes, but questions remain [39]. The absorption of bovine bone HA is active but seems to be very slow. Indeed, the material is degraded more slowly than it is resorbed [40,41,42]. A bone biopsy after alveolar ridge augmentation confirms that particles of bone graft substitute of bovine origin can be found up to 10 years after the procedure. Therefore, xenografts are considered as nonabsorbable biomaterials in daily practice [43]. 

The term alloplastic bone grafts refers to synthetic biomaterials. Synthetic materials currently exhibit only osteoconductive properties. Materials that belong in this category include metals, polymers, polyglycolides, calcium phosphate cements, HA, tricalcium phosphate (TCP), and bioglass [20,44]. The biocompatibility/histocompatibility and osteoconductivity of these alternatives are advantageous. In addition, no donor site is required and there is no risk of infectious disease transmission [45,46,47]. The efficacy of alloplastic biomaterials depends on maintaining the space for new bone formation and the rate of their resorption. Many of the alloplastic materials are resorbed slowly or not at all, which is one of the disadvantages. New bone requires the space originally occupied by the bone graft. However, if the bone material is not resorbed, the available space for bone formation is limited, reducing the overall volume of newly formed bone [48,49]. Synthetic calcium phosphates which are most commonly used in dentistry consist either of solid HA, β-tricalcium phosphate (β-TCP), or a mixture of the two called biphasic (two-phase) calcium phosphates (BCP), composed of 16.5% biphasic granules and 83.5% nano-HA gel, which was used in our study [28,50]. HA is very slowly resorbed, therefore serving as a support to maintain the integrity and completeness of the defect due to its osteoconductivity, whereas β-TCP is much more quickly resorbed and contributes to the formation of new bone by releasing calcium and phosphorus ions [50,51].

Also, after tooth extraction, the supply of metabolic products is interrupted. Immune cells produce various cytokines that are temporally and spatially controlled at the site of injury and cause acute inflammation, angiogenesis, and accumulation of mesenchymal cells. The recruited osteoprogenitor cells produce bone morphogenetic protein 2 (BMP-2), which, in coordination with other factors, promotes local accumulation and osteogenic differentiation of mesenchymal cells at the site of injury [52,53,54,55,56]. Mesenchymal cells differentiate into chondrocytes and osteoblasts and proliferate until they fully differentiate into a mature hypertrophic phenotype [57,58,59]. Several transcription factors control the differentiation of osteoblasts, which are responsible for synthesis and mineralization of the bone matrix. Osterix (Osx) has been identified as the most highly expressed transcription factor in the final stages of osteoblast differentiation in newly formed bone and induces the formation of collagen, osteocalcin (OCN), and osteopontin (OPN), promoting bone remodeling [60,61,62].

The main aim of this study was to determine the healing ability (histologically and immunohistologically) of alveolar bone defects during augmentation with two different biomaterials: injectable biphasic calcium phosphate (BCP) and anorganic bovine bone (ABB). The ability of BSB to restore damaged tissue was quantitatively and qualitatively evaluated, and pathohistological changes were described on bone biopsies six months after augmentation. In addition, the expression of Osx and BMP-2 in bone remodeling and mesenchymal cell differentiation was detected by immunohistochemistry.

## 2. Results

### 2.1. Demographic Data

The study included 38 participants, of whom 17 were males (45%) and 21 were females (55%). They were divided into two groups: the test group and the control group. The mean age of the respondents was 35.03 ± 9.16 years (female = 36.67 ± 6.39; male = 33.00 ± 11.61). A brief description of the characteristics of the sample itself can be found in Table 1.

### 2.2. Quantitative Analysis of the Histological Bone Biopsy Samples

In the quantitative analysis, the areas of newly formed bone, residual BSB, and soft tissue structures were determined with respect to the total area of the histological sample in the field of view. After processing all the results, the obtained areas were converted into volume percentages (%), as shown in Table 2.

Since the requirements for performing parametric tests were met, the *t*-test was used to compare the small samples. The *t*-test was used to compare each variable separately, i.e., newly formed bone (*t* (38) = 0.487; *p* = 0.629), residual biomaterial (*t* (38) = 0.705; *p* = 0.485), and soft tissue (*t* (38) = −1.626; *p* = 0.113), as shown in Table 3.

As shown by the *t*-test values, there is no statistically significant difference between the test group and the control group in terms of newly formed bone, residual biomaterial, and soft tissue.

### 2.3. Qualitative Histological Analysis

In the qualitative analysis, the pathohistological response of the host tissue to the used BSB was evaluated, i.e., osteoblasts, osteocytes, fibroblasts, fibrocytes, blood vessels, and cells of the monocyte–macrophage system were described. In addition, the newly formed bone, the residual biomaterial, and the soft tissue were described.

#### HE histological Staining

**Figure 1:**
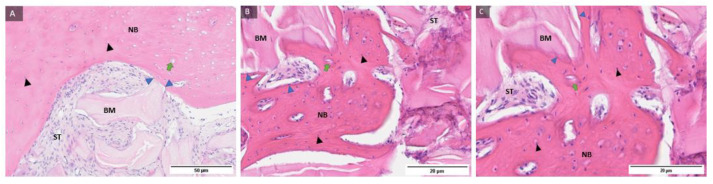
Examples of preparations of the control group (**A**–**C**) with labeled newly formed bone (NB), residual biomaterial (BM), soft tissue (ST), osteoblasts (blue filled triangle), osteocytes (black filled triangle), and Howship lacunae in the bone bed with a blood vessel (green arrow). Magnification: 100, 200, 400×.

Representative samples of the control group and histological staining with HE under magnification of 100, 200, and 400 times and labelling of newly formed bone, residual biomaterial, soft tissue, osteoblasts at the boundary between newly formed bone and residual biomaterial, osteocytes in newly formed bone and Howship lacunae in the bone bed with a blood vessel indicating the integration of biomaterials and newly formed bone (Figure 1). 

**Figure 2:**
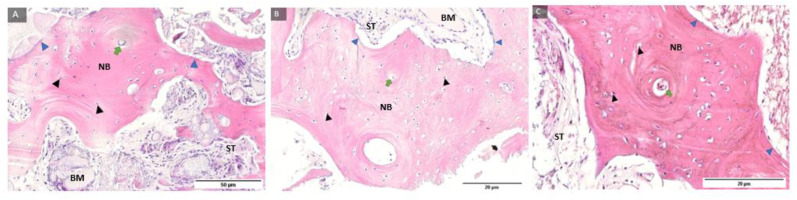
Examples of preparations of the test group (**A**–**C**) with labeled newly formed bone (NB), residual biomaterial (BM), soft tissue (ST), osteoblasts (blue filled triangle), osteocytes (black filled triangle), and Howship lacunae in the bone bed with a blood vessel (green arrow). Magnification: 100, 200, 400×.

Representative samples of the test group and histological staining with HE under magnification of 100, 200, and 400 times and labeling of newly formed bone, residual biomaterial, soft tissue, osteoblasts at the boundary between newly formed bone and residual biomaterial, osteocytes in newly formed bone and Howship lacune in the bone bed with a blood vessel indicating the integration of biomaterials and newly formed bone (Figure 2).

**Figure 3:**
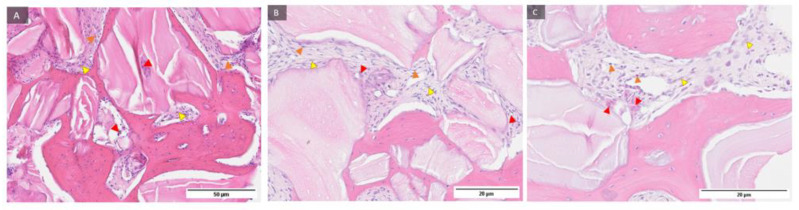
Examples of preparations of the control group (**A**–**C**) with labelled fibroblasts (yellow filled triangle), fibrocytes (orange filled triangle), and cells of the monocyte–macrophage system (red filled triangle). Magnification: 100, 200, 400×.

Representative samples of the control group and histological staining with HE under magnification of 100, 200, and 400 times and labeled fibroblasts and fibrocytes in the soft tissue and cells of the monocyte–macrophage system around the residual biomaterial, indicating biomaterial degradation (Figure 3).

**Figure 4:**
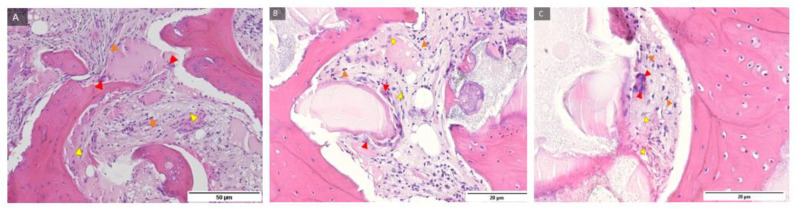
Examples of preparations of the test group (**A**–**C**) with labelled fibroblasts (yellow filled triangle), fibrocytes (orange filled triangle), and cells of the monocyte–macrophage system (red filled triangle). Magnification: 100, 200, 400×.

Representative samples of the test group and histological staining with HE under magnification of 100, 200, and 400 times and labelled fibroblasts and fibrocytes in the soft tissue and cells of the monocyte–macrophage system around the residual biomaterial, indicating the decomposition of the biomaterial (Figure 4).

### 2.4. Immunohistochemical Analysis

Immunohistochemical analysis of bone biopsy samples was performed to detect Osx transcription factor and BMP-2 protein.

**Figure 5:**
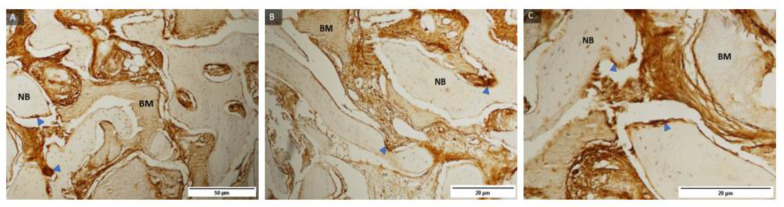
Examples of preparations of control group (**A**–**C**) and Osx immunohistochemical staining of bone sections after implantation of transcription factor Osx with labeled newly formed bone (NB), residual biomaterial (BM), and cells with 3 (+++) Osx expression (triangle marked in blue). 100, 200 and 400× magnification.

Representative samples of control group and Osx immunohistochemical staining at magnification of 100, 200, and 400 times (Figure 5) showed newly formed bone, residual biomaterial, and cells with 3 (+++) Osx expression in pre-osteoblasts anchored at the margin of newly formed bone, indicating their transition into mature osteoblasts and osteocytes. Trabecularization of the new bone was also observed, indicating continuous remodeling.

**Figure 6:**
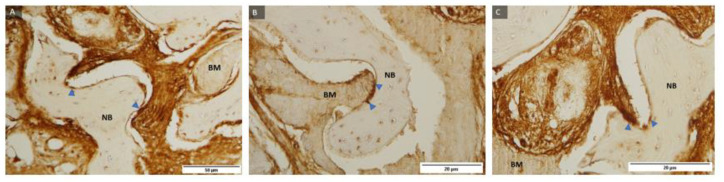
Examples of preparations of the test group (**A**–**C**) and immunohistochemical Osx staining of bone sections after implantation of transcription factor Osx with labelling of newly formed bone (NB), residual biomaterial (BM), and cells with a strength of 3 (+++) expression of Osx (blue marked triangle). 100, 200, and 400× magnification.

Representative samples of the test group and immunohistochemical Osx staining under magnification of 100, 200, and 400 times (Figure 6) and labeled newly formed bone, residual biomaterial, and cells with a strength of 3 (+++) expression of Osx in pre-osteoblasts anchored at the boundary of the newly formed bone, indicating their transition into mature osteoblasts and osteocytes. Trabecularization of the new bone was also observed, indicating continuous remodeling.

**Figure 7:**
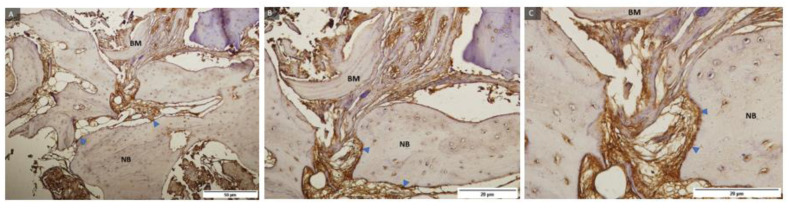
Examples of preparations of the control group (**A**–**C**) and BMP-2 immunohistochemical staining of bone sections after implantation of BMP-2 protein with labeled newly formed bone (NB), residual biomaterial (BM), and cells with expression level 3 (+++) BMP- 2 (blue marked triangle). 100, 200, and 400× magnification.

Representative samples of control group and BMP-2 immunohistochemical staining under magnification of 100, 200, and 400 times (Figure 7) and labelled newly formed bone, residual biomaterial, and cells with expression level 3 (+++) BMP-2 present mainly in zones where differentiation of mesenchymal cells into pre-osteoblasts continued, indicating regeneration of damaged tissue.

**Figure 8:**
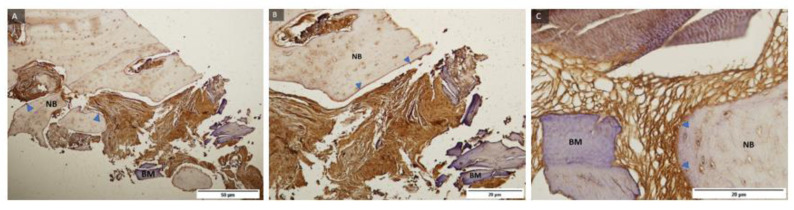
Examples of preparations of the test group (**A**–**C**) and BMP-2 immunohistochemical staining of bone sections after implantation of BMP-2 protein with labelling of newly formed bone (NB), residual biomaterial (BM), and cells with expression level 3 (+++) BMP-2 (triangle marked in blue). 100, 200, and 400× magnification.

Representative samples of the test group and BMP-2 immunohistochemical staining under magnification of 100, 200, and 400 times (Figure 8) and labelled newly formed bone, residual biomaterial, and cells with expression level 3 (+++) BMP-2 present mainly in zones where differentiation of mesenchymal cells into pre-osteoblasts continued, indicating regeneration of damaged tissue.

## 3. Discussion

This study included 38 subjects, 17 (45%) male and 21 (55%) female. They were divided into two groups, the test group and the control group. The mean age of the respondents was 35.03 ± 9.16 years (females = 36.67 ± 6.39; males = 33.00 ± 11.61). Parallels can be drawn with human studies conducted by Čandrlić et al. [63], Jelušić et al. [64], and Cordaro et al. [65]. The study by Čandrlić et al. investigated the qualitative and quantitative effects of injectable BCP and other types of xenograft in the technique of alveolar preservation. That study included 40 participants, 15 (37.5%) male and 25 (62.5%) female. The study by Jelušić et al. investigated the qualitative and quantitative effects of pure single-phase ß-TCP and granulated BCP in the sinus floor lifting technique; it involved 43 subjects, 53.3% male and 46.7% female. The study by Cordaro et al. also investigated the effects of granulated BCP and other types of xenografts in the sinus floor lifting technique; it involved 37 subjects. These three studies, even though they used different xenografts and alloplastic BSBs, can confirm a study structure similar to those in which the lowest age of the subjects was 18 years and the main inclusion and exclusion criteria regarding indications and contraindications for implant therapy were confirmed in all studies. The bone biopsy was taken at the site of the future implant six months after the augmentation procedure, which is consistent with this and many other studies.

Quantitative and qualitative analysis of bone biopsy samples is used to evaluate regenerated augmentation areas, which is supported by many previous studies in addition to ours [66,67,68]. The results of the comparison of the observed structures in the control group, in which the xenograft (Bio-oss^®^) was used, were expressed as mean ± standard deviation of the mean and consisted of the following: newly formed bone (41.73 ± 13.99%), residual biomaterial (31.72 ± 15.52%), and soft tissue structures (26.54 ± 7.25%). On the other hand, the pathohistological response of the host tissue to the used BSB was evaluated by qualitative analysis.

In the literature, Bio-Oss^®^ is one of the best-documented bovine xenografts and has been used as a control group in many studies [65,69,70]. Following the currently available literature on animal studies, such as the study by Jensen et al. in 1996, [71] for the first time a quantitative and qualitative analysis described the formation of newly formed bone and the ability of BSB to restore damaged tissue stimulated by the use of xenograft (Bio-Oss^®^); the effect of this was investigated in the control group in this study. This is the case with studies such as that of McAllister et al. [72,73]: In two studies of chimpanzees 7.5 months after augmentation, the percentage of newly formed bone was 47% and 62%, while the percentage of residual BSB was 19% in both studies. Mah et al. and Scarano et al. [74,75] described percentages of newly formed bone of 47.4 ± 7.1% and 39 ± 3.3% in their studies of animal models of rat calvaria between 112 and 168 days. Similarly, in a more recent study of pigs performed by Aludden et al. in 2020 [76], the percentage of newly formed bone 20 weeks after the augmentation procedure was 60%, whereas the residual BSB was 25%. These results and the follow-up period roughly correlate with these results, regardless of the animal models and sample size mentioned.

According to the literature on human studies using this form of xenograft six months after augmentation, an older study by Zitzmann et al. in 2001 [77] confirmed 36.7 ± 26.6% newly formed bone, 30.5 ± 4.6% residual BSB, and 37.6 ± 20.5% soft tissue structures, which is similar to the results obtained in this study. A more recent study by Amoian et al. [78] can also be related to this control group in terms of newly formed bone, which was 38.66% in this study, although the sample consisted of only six patients. On the other hand, the study by Scarano et al. [79], although performed on a much larger number of subjects, confirmed the regenerative effect of Bio-Oss^®^ in terms of newly formed bone with 39 ± 1.6%, 31 ± 1.4%, residual BSB, and 34 ± 1.6% soft tissue structures, which is the closest to the results obtained in this study six months after augmentation with the same BSB. It is interesting to mention the recent study by Wei et al. [80] investigating the effect of Bio-Oss^®^ in socket preservation after molar extraction in patients with periodontitis. Although such patients were excluded in our study, the study by Wei. et al. showed promising results in terms of an increase in alveolar bone dimensions in the maxilla and mandible 6 months after augmentation. These conclusions confirm a good regenerative potential of Bio-Oss^®^ in alveolar bone regeneration as shown in our study. Conversely, some human studies have shown that alveolar ridge defects grafted with Bio-Oss^®^ resulted in different percentages of newly formed bone, residual BSB, and soft tissue structures from the above results, such as the studies by Froum et al. [81], Schmitt et al. [82], Lorenz et al. [83], Fienitz et al. [84], etc. Moreover, studies performed in recent years indicate different proportions of the variables already mentioned, as in the studies by Sivolella et al. [85], Pignaton et al. [86], and Santos et al. [87]. Such results can be explained by different initial morphology of the defect, type of wound closure, elevation of the flap, use of different membranes, location of the biopsy, and different follow-up periods.

In addition to histomorphometric analysis, the interest of previous studies, such as this one, focused on the interactions between BSB and host tissues, such as the biological response of the tissue related to the origin of BSB, which is crucial for qualitative analysis. Notwithstanding the slow resorption demonstrated in many studies, the qualitative light microscopic analyses in other studies as well as this study strongly demonstrate that the sites treated with Bio-Oss^®^ show good integration between the biomaterial and the surrounding host tissue [82,88]. Furthermore, after a period of six months, osteoblasts at the boundary between newly formed bone and residual biomaterial, osteocytes in the newly formed bone, and Howship lacunae in the bone bed with a blood vessel indicate the integration of biomaterial. Moreover, newly formed bone can be observed in these samples. On representative samples of histological staining, cells of the monocyte–macrophage system are also observed around the residual biomaterial, indicating biomaterial degradation, which can be related to many other studies. For example, Piattelli et al., after a certain follow-up period on histological samples, demonstrated the presence of cells of the monocyte–macrophage system around the aforementioned BSB, indicating its slower resorption [43,89]. According to some authors, such behavior in vivo could be partially prevented by a specific high-temperature treatment of BSB in the course of the production process. This treatment alters the mineral structure of bone, so that the resulting BSB usually has a reduced resorption potential [38]. These findings correlate with the results obtained and indicate that more residual biomaterial remained in the control group (Bio-Oss^®^—31.72 ± 15.52%) than in the test group (maxresorb inject^®^—28.61 ± 11.38%) and that xenograft resorption was ultimately slower. Bio-Oss^®^ has proven to be a valuable alternative to the gold standard from a clinical point of view, ensuring good quality of the newly formed bone and promising a long-term regeneration rate [90].

Injectable BCP (maxresorb^®^ inject) with a composition of 16.5% biphasic granules and 83.5% nano-HA gel, representative of the group of alloplastic BSB, was used in the test group. The quantitative results of this study, i.e., the comparison of newly formed bone, residual BSB, and soft tissue structures in the test group in which injectable BCP (maxresorb^®^ inject) was used, are also given as mean ± standard deviation of the mean and were as follows: newly formed bone (39.91 ± 8.49%), residual biomaterial (28.61 ± 11.38%), and soft tissue structures (31.49 ± 11.09%).

From the currently available literature, Gauthier et al. [91] were among the first to perform an animal study on the use of injectable synthetic BSB at a ratio of 60/40 HA/ß-TCP and their ability to induce new bone formation in dogs. After three months of augmentation, 48.96 ± 8.90% new bone was formed in the study. From the preliminary histomorphometric results of an animal study, it can be concluded that an alloplastic BSB with injectable capabilities in the ratio 60/40 HA /ß-TCP stimulates the formation of new bone tissue. The same authors showed in a 2004 study of dogs that three months after implantation of injectable BCP, the number of newly formed bone significantly exceeded the number of unfilled defects [92]. This was also confirmed by the study by Aral et al. [93] using BSB in injectable form. Moreover, in a slightly later study by Struillou et al. [94] using the injectable form of BCP, the percentage of newly formed bone was 35.5 ± 13.9%, which is very similar to the results obtained in this study. Three months after augmentation is a rather short period of time to detect biomaterial deterioration; accordingly, the authors mentioned above concluded at that time that long-term studies would be helpful to assess the biodegradation behavior of biomaterials. In order to obtain the most accurate and relevant results, histological samples were also taken from this group six months after augmentation.

The main evidence of regenerative potential and formation of the new bone is found in the current literature on human studies using the injectable form of BCP, but it is quite limited. Histologic and histomorphometric analyses of bone biopsy samples taken four and six months after augmentation, in the studies by Papanchev et al. [95] and Lorenzo et al. [96], indicated equal amounts of newly formed bone and soft tissue. Contrary to recommendations, bone biopsy samples were taken from 21 patients four months after augmentation in the study by Lorenzo et al. However, they showed a percentage of newly formed bone of 44.92 ± 5.16%, which also correlates with this study, in contrast to the residual biomaterial, which was 2.59 ± 2.05% in this study, and soft tissue structures of 52.49 ± 6.43%. In the previously mentioned study by Čandrlić et al. [63] using injectable BCP in combination with another xenograft, the percentage of newly formed bone was 26.47 ± 14.72%, residual biomaterial 13.1 ± 14.07%, and soft tissue structures 60.43 ± 12.73%. Regardless of the different results compared with this study, the regenerative potential of injectable BCP was demonstrated in both studies. It is also important to mention some recent studies using BCP with a composition of 60/40 HA /β-TCP in granular form, which showed similar results to this study with injectable BCP six months after augmentation. For example, in the study by Jelušić et al. [64] in 30 patients, the percentage of newly formed bone was 38.42 ± 61%; in the study by Nery et al. [97] in 10 patients, it was 43.4 ± 6.1%; and in the study by Flichy-Fernanadez et al. [98] in 16 patients, it was 34.09 ± 14.11%. These quantitative results confirm the osteoconductive potential of BCP.

Qualitative analysis was also used in this group to evaluate the pathohistological response of the host tissue to the BSB used. Alloplastic BSBs enhance growth and proliferation in vivo and stimulate osteoblasts to deposit mineralized extracellular matrix as a structural scaffold for osteogenic cell migration [99,100]. The geometry, ultrastructure, and mechanical properties of these BSBs, in addition to their chemical composition, are critical for effective bone defect repair, resorption, and concomitant replacement with newly formed bone [101]. Studies by Khaled et al. [102] and Georgiev et al. [103] combining injectable BSB with HA nanoparticles found that HA in the form of smaller granules promoted better cell contact, leading to faster biomaterial resorption and new bone formation. This was also confirmed in this study, in which BSB was used with the addition of HA. Histological analysis showed that the BSB particles were integrated and gradually replaced by newly formed bone. On the other hand, the resorption of BSB can be explained by the fact that the aqueous part of the gel dissolves immediately after insertion, leaving behind nano-HA and HA /ß-TCP particles. As known from the instructions for use of this BSB and from some studies such as that by Gotz et al. [104], nano-HA particles show high biological activity due to their large surface area. The nanoporosity of biomaterials appears to facilitate the uptake of bone-specific molecules and growth factors such as alkaline phosphatase, BMP-2, collagen type I, osteocalcin, and osteopontin. This in turn facilitates the recruitment of osteoblast precursors and their differentiation into mature osteoblasts by means of the monocyte–macrophage cell adhesion system, ultimately leading to the gradual resorption of BSB and the formation of mature bone tissue. All the mentioned cells and structures are also described in the representative samples of this study. It is important to note that the samples in both groups showed no signs of an inflammatory response, while cells of the monocyte–macrophage system, indicating resorption, were detected only at the margin of BSBs.

Immunohistochemical analysis of the control and test groups was used to detect the transcription factor Osx and BMP-2 protein, which play a role in bone remodeling and the differentiation of mesenchymal cells. The immunohistochemical findings with expression level (Osx and BMP-2) were reviewed and evaluated semiquantitatively. Intensity level 3 (+++), i.e., strong staining indicating good osteoconductive properties of both BSBs, was detected in both groups. Representative samples from the control and test group and Osx immunohistochemical staining showed cells with Osx expression of intensity level 3 (+++) in pre-osteoblasts anchored to the margin of newly formed bone, indicating their transition into mature osteoblasts and osteocytes. In addition, trabecularization of the new bone was observed, indicating continuous remodeling. Osx has been shown to be involved in osteoblast differentiation, maturation, and activity; it regulates the expression of various markers, i.e., osteoblast proteins, the most important of which are osteopontin (OPN) and osteocalcin (OCN), etc., as indicated by the currently available literature. Previous studies have shown that Osx is an essential transcription factor in osteogenic differentiation [105,106].

On the other hand, representative samples from the control and test groups and BMP-2 immunohistochemical staining showed cells with BMP-2 expression levels of 3 (+++), present mainly in the zones where differentiation of mesenchymal cells into pre-osteoblasts continued, indicating recovery, i.e., regeneration of the damaged tissue. According to the literature, BMP-2 is a protein that acts as a potent osteogenic factor and promoter of osteoblast differentiation, which was expressed in similar studies with this type of BSB, confirming the results obtained [104,107].

## 4. Materials and Methods

### 4.1. Subjects 

Thirty-eight subjects participated in this study. The main criteria for enrollment were that the patients had at least one tooth scheduled for extraction, all previous therapeutic options had been exhausted, and they had the possibility of dental implant placement at the extraction site after alveolar ridge augmentation. The criteria for inclusion were (1) age of subjects ≥18 and ≤60 years, (2) understanding of the protocol and informed consent signed by each subject, and (3) satisfactory physical and mental health of the subjects. Subjects who had some of the exclusion criteria were excluded from the study: (1) at least one absolute contraindication to implant therapy described by Wang and Hwang 2006. [108]; (2) subjects with systemic diseases such as osteoporosis, osteopenia, uncontrolled diabetes, vitamin D deficiency, bisphosphonate therapy, glucocorticoid therapy, hypothyroidism, uncontrolled cardiovascular diseases (hypertension, coronary artery disease, heart failure), and local factors: consumption of tobacco products (more than 10 cigarettes per day), poor oral hygiene; (3) patients with untreated periodontitis, patients with acute odontogenic infection, patients with periapical lesion, and patients who had previously received BSB at the extraction site. In addition, pregnant and lactating women were not included. This study was approved by the Ethics Committee of the Osijek-Baranja County Health Center. In the treatment of all patients, the Declaration of Helsinki of the International Medical Association—1964 (most recent update—2013) was fully observed [109].

### 4.2. Surgical Phase

**Figure 9:**
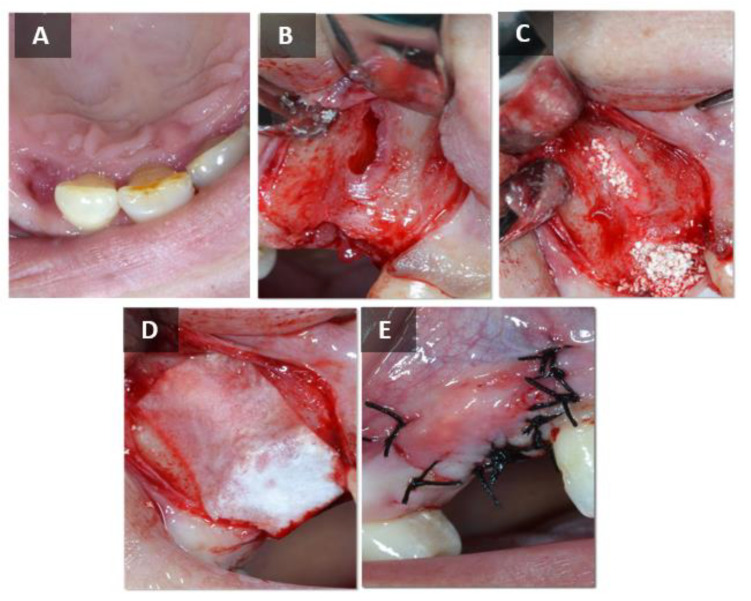
Surgical procedure—control group. (**A**) Hopeless tooth. (**B**) Alveolus and bone defect after tooth extraction. (**C**) Placement of the BSB. (**D**) Placement of the resorptive membrane. (**E**) Sutured wound.

**Figure 10:**
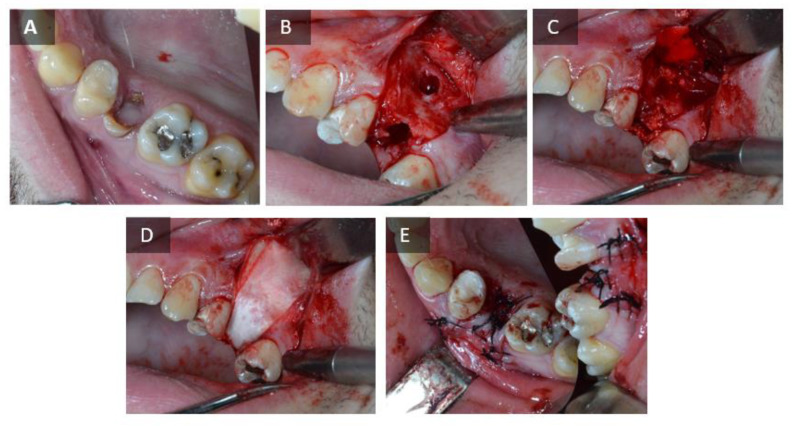
Surgical procedure—test group. (**A**) Hopeless tooth. (**B**) Alveolus and bone defect after tooth extraction. (**C**) Placement of the BSB. (**D**) Placement of the resorptive membrane. (**E**) Sutured wound.

Before the start of the procedure, radiographs were taken at the planned tooth ex-traction sites. Patients who met the inclusion criteria were prescribed an antibiotic (amoxicillin 500 mg, Belupo, Koprivnica or clindamycin-MIP 600 mg, Chem.-pharm. Fabrik GmbH, Ingbert, Germany, in case of allergy to the penicillin group of antibiotics) one hour before the procedure; local anesthesia with 4% articaine and epinephrine 1:100,000 (Ubistesin forte^®^, 3M Deutschland GmbH, Neuss, Germany) was administered. Patients who agreed to participate in the study were randomized into two groups. The first group consisted of patients who received the test BSB (maxresorb^®^ inject, botiss biomaterials, Zossen, Germany), and the second group consisted of patients who received an alternative to the gold standard, ABB (Bio-Oss^®^, Geistlich-Pharma, Wolhunsen, Switzerland). The socket was filled with injectable BCP in the test group and with ABB in the control group. Finally, after filling the defect, an absorbable collagen membrane (Jason^®^ membrane, botiss biomaterials, Zossen, Germany) was fitted to the surgical wound in both groups and the mucoperiosteal flap was closed with nonabsorbable 5.0 monofilament suture (Sofsilk™, Covidien, Dublin, Ireland). The surgical procedure described is shown in Figure 9 and Figure 10. The patient was prescribed an analgetic (ibuprofen 600 mg, Belupo, Koprivnica or paracetamol 500 mg, Lek Pharmaceu-ticals d.d., Ljubljana, Slovenia, if allergic to ibuprofen). The patients took the remaining dose of antibiotics for the next 7 days and 0.2% chlorhexidine solution (Curasept ADS^®^ 220, Saronno, Italy) for postoperative care of the oral cavity. One week after augmentation, the patient was referred for a radiological examination of the augmented area by cone beam computed tomography (CBCT) to verify the stability of the BSB at the augmentation site. Before completion of the six months of healing, thepatient was invited to obtain a control CBCT scan, which measured the dimensions of the alveolar ridge at the site of implantation. Immediately thereafter, the second phase of the study was scheduled, i.e., biopsy of the alveolar bone with final placement of a dental implant.

The trepan drill (Komet Dental, Gebr. Brasseler GmbH & Co. KG, Lemgo, Germany) used to take the bone biopsy sample had a smaller inner diameter (2.5 mm) than the final drill from the standardized set for shaping and bed preparation of the dental implant (Ankylos, Denstply Sirona Implants, Mannheim, Germany). From an ethical point of view, this avoids excessive removal of healthy bone. The bone biopsy samples were then left in a 4% formaldehyde solution (BioGnost Ltd., Zagreb, Croatia) and sent to the laboratory for histological and immunohistochemical analysis.

### 4.3. Qualitative Analysis of the Histological Bone Biopsy Samples Was Performed

A standardized protocol for histological preparation of mineralized bone samples was applied to the samples, which included the following: fixation in a 4% formaldehyde solution (BioGnost Ltd., Zagreb, Croatia), dehydration in increasing alcohol concentrations (75%, 85%, 95%, and finally 100%), decalcification with ethyldiaminetetraacetic acid (EDTA, Osteomoll^®^, Sigma-Aldrich, St. Louis, MO, USA) for two weeks, embedding in paraffin blocks, and sectioning. Six contiguous sections with a thickness of 5 μm were prepared with a microtome (SLEE, Mainz, Germany) and placed on slides after drying. Before staining, the tissue had to be rehydrated by placing it twice in xylene (BioGnost Ltd., Zagreb, Croatia) for 15 min each, then in a descending series of alcohol (100%, 95%, 85%, and finally 75%), and finally in distilled water. The tissue was then stained with hemalaun-eosin (HE) using histological staining kits. Finally, dehydration was repeated in an increasing series of alcohols (75%, 85%, 95%, and finally 100%). Digital photomicrographs were taken using a light microscope (Leica DMRB, Leica Microsystems GmbH, Wetzlar, Germany) with an attached video camera (Axio Imager M2, Zeiss, Oberkochen, Germany) at magnification of 10, 20, and 40 times. In a qualitative analysis, the pathohistological response of the host tissue to the BSB used was evaluated, i.e., osteoblasts, osteocytes, fibroblasts, fibrocytes, blood vessels, and cells of the monocyte-macrophage system were described.

### 4.4. Quantitative Analysis of Histological Bone Biopsy Samples

Quantitative histological analysis of bone biopsies taken after six months of healing was performed on the same samples previously prepared for pathohistological analysis. The digital photomicrographs were stored in an uncompressed format used to store high-resolution images until analysis. The digital photomicrographs were loaded into the free ImageJ computer program (Wayne Rasband, National Institute of Health, Bethesda, MD, USA). All the photomicrographs were taken under the same conditions (magnification of 100, 200, and 400 times, PNG format). Before starting the analysis, parameters such as setting the scale on the basis of the known distance and converting it to the unit of length (μm) were set. Also, the possibility of manual correction was set to exclude from the analysis any edge regions that were not completely clear or that represented an artifact. Each sample was analyzed individually such that the digital micrograph was adjusted using the threshold option, with manual manipulation allowing areas of interest (ROI) to be marked in different colors. Accordingly, the areas of newly formed bone, residual BSB, and soft tissue structures were determined in relation to the total area of the histological sample in the field of view, and after all the results were processed, the obtained areas were converted into volume percentages (%). All the preparations were additionally examined by two independent researchers.

### 4.5. Immunohistochemical Analysis of Bone Biopsy Samples

Immunohistochemical analysis was performed on four histological bone biopsy samples to detect Osx transcription factor and BMP-2 protein. Bone tissue samples with a thickness of 5 μm were deparaffinized in xylene (BioGnost Ltd., Zagreb, Croatia) and then rehydrated in alcohol of decreasing concentration (100%, 95%, 85%, and finally 75%). The next step was protein renaturation, which was achieved by incubating the slices in citrate buffer (10 mM sodium citrate, 0.05% Tween 20, pH 6.0) in a water bath at 90° for 10 min. After cooling, the samples were washed in a physiological solution buffered with phosphate-buffered saline (PBS) at pH 7.2. Blockade of endogenous peroxidase activity to avoid nonspecific binding was performed with 0.3% hydrogen peroxide H_2_O_2_ (Merck, Darmstadt, Germany), followed by a 10-min wash in PBS. According to the manufacturer’s recommendations, the samples were incubated overnight at 4 °C with a rabbit polyclonal antibody to Sp7/Osx (ab229258, Abcam, Cambridge, UK) and a rabbit polyclonal antibody to BMP-2 (ab14933, Abcam, Cambridge, UK). Table 4 provides information on the antibodies and incubation procedures. At room temperature, the secondary antibody was incubated for 45 min. Then, 3,3’-diaminobenzidine (DAB, DakoCytomation, Glostrup, Denmark) and peroxidase-conjugated streptavidin (LSAB + kit, DakoCytomation, Glostrup, Denmark) were added for visualization. The samples were then purified with distilled water, filtered, and stained with hemalun-eosin. The slides were mounted on medium (Biomount, Biognost, Zagreb, Croatia) and analyzed using a light microscope (Olympus, Tokyo, Japan) and a digital camera (Sony, Tokyo, Japan). Immunohistochemical findings with the level of expression (Osx and BMP-2) were evaluated semiquantitatively by an experienced pathologist, who assigned a value of 0–3 “plus points” depending on the intensity of staining in the following manner: 0 = negative; 1 = weak staining (+); 2 = moderate staining (++); 3 = strong staining (+++). All the preparations were additionally examined by two independent researchers.

### 4.6. Statistical Methods

For statistical analysis, IBM SPSS (version 24, IBM Corporation, Armonk, NY, USA) was used. The results of the Shapiro–Wilk test were used to determine whether the distribution was normal. The mean and standard deviation of the mean were used for all results. A *t*-test was used to evaluate the significance of the difference between the two samples in the context of a normal distribution. Any *p* value less than 0.05 was considered significant.

## 5. Conclusions

Histopathologic, histomorphometric, and immunohistochemical analyzes of these two BSBs showed comparable results and proved that maxresorb^®^ inject might be as suitable and successful as BSB for alveolar ridge augmenatation. It is necessary to highlight the strengths of this study. The study was conducted as a randomized controlled human clinical trial comparing quantitative, qualitative, and immunohistochemical analysis of two BSBs. The histomorphometric, histological, and immunohistochemical analysis and the use of a standardized free program with a detailed description of the samples allowed the methodology to be reproduced and the results obtained to be compared with future studies. All the subjects in the study underwent a standardized protocol in terms of surgical procedures and time from bone augmentation to biopsy. However, due to the small number of samples processed, especially for immunohistochemical analysis, further clinical studies with a larger sample and a longer follow-up period could be used to draw definitive conclusions in this regard. Notwithstanding some methodological limitations, the comparable results obtained with injectable BCP compared to the gold standard alternative, i.e., ABB, represent a promising outcome for the purpose of alveolar ridge augmentation after tooth extraction and dental implant placement.

## Figures and Tables

**Table 1:** Comparison of test and control groups by average age of male and female.

	Male	Female
Control group ^1^	33.54 ± 10.08	35.58 ± 7.48
Test group ^2^	32.60 ± 13.10	38.11 ± 4.60
Total	33.00 ± 11.61	36.67 ± 6.39

^1^ Anorganic bovine bone, ^2^ Injectable biphasic calcium phosphate.

**Table 2:** Minimum and maximum values of newly formed bone, residual biomaterial, and soft tissue between the control and test groups, expressed in percentages.

	NB ^1^		BM ^2^		ST ^3^	
	Min	Max	Min	Max	Min	Max
Control group	15.05%	68.61%	7.58%	65.62%	12.76%	38.86%
Test group	24.70%	51.77%	4.66%	50.04%	19.24%	55.00%

^1^ Newly formed bone, ^2^ Residual biomaterial, ^3^ Soft tissue.

**Table 3:** Comparison of the tested and control groups according to variables, expressed as percentages.

	NB ^1^	BM ^2^	ST ^3^
Control group ^1^	41.73 ± 13.99%	31.72 ± 15.52%	26.54 ± 7.25%
Test group ^2^	39.91 ± 8.49%	28.61 ± 11.38%	31.49 ± 11.09%
*p*-value *	*p* = 0.629	*p* = 0.485	*p* = 0.113

^1^ Anorganic bovine bone, ^2^ Injectable biphasic calcium phosphate, ^3^ Soft tissue, * *t*-test.

**Table 4:** Antibodies used for immunohistochemical analysis.

Antibody	Isotype	Manufacturer	Incubation
Anti-Sp7/Osx ^1^	Rabbit polyclonal	Abcam, Cambridge, UK	1:200, overnight, 4°
Anti-BMP-2 ^2^	Rabbit polyclonal	Abcam, Cambridge, UK	1:200, overnight, 4°

^1^ Osterix, ^2^ Bone morphogenetic protein 2.

## Data Availability

The data presented in this article are available on request from the corresponding authors.
